# RANK signaling in osteoclast precursors results in a more permissive epigenetic landscape and sexually divergent patterns of gene expression

**DOI:** 10.7717/peerj.14814

**Published:** 2023-02-09

**Authors:** Abigail L. Keever, Kathryn M. Collins, Rachel A. Clark, Amber L. Framstad, Jason W. Ashley

**Affiliations:** 1 Elson S. Floyd College of Medicine, Washington State University, Spokane, WA, United States; 2 Department of Biology, Eastern Washington University, Cheney, WA, United States

**Keywords:** Osteoclasts, RANK signaling, RNA-seq, ATAC-seq, Sexual dimorphism, Macrophages

## Abstract

**Background:**

Sex is an important risk factor in the development of osteoporosis and other bone loss disorders, with women often demonstrating greater susceptibility than men. While variation in sex steroids, such as estradiol, accounts for much of the risk, there are likely additional non-endocrine factors at transcriptional and epigenetic levels that result in a higher rate of bone loss in women. Identification of these factors could improve risk assessment and therapies to preserve and improve bone health.

**Methods:**

Osteoclast precursors were isolated male and female C57Bl/6 mice and cultured with either MCSF alone or MCSF and RANKL. Following the culture period RNA was isolated for RNA sequencing and DNA was isolated for tagmentation and ATAC sequencing. RNA-Seq and ATAC-seq were evaluated *via* pathway analysis to identify sex- and RANKL-differential transcription and chromatin accessibility.

**Results:**

Osteoclasts demonstrated significant alterations in gene expression compared to macrophages with both shared and differential pathways between the sexes. Transcriptional pathways differentially regulated between male and female cells were associated with immunological functions with evidence of greater sensitivity in male macrophages and female osteoclasts. ATAC-Seq revealed a large increase in chromatin accessibility following RANKL treatment with few alterations attributable to sex. Comparison of RNA-Seq and ATAC-seq data revealed few common pathways suggesting that many of the transcriptional changes of osteoclastogenesis occur independently of chromatin remodeling.

## Introduction

Osteoclasts are multinucleated hematopoietic-lineage cells responsible for the dissolution of bone matrix required for both physiological bone growth and remodeling (Teitelbaum, 2007). While indispensable for normal bone homeostasis, excessive osteoclastic resorption can lead to insufficient bone mineral density, joint destruction, tooth loss, and fracture (Ensrud & Crandall, 2017). Multiple genetic and environmental factors contribute to overall risk of pathologically elevated osteoclast activity, but the greatest contributors are advanced age and female sex (Watts et al., 2008; Johannes de Villiers, 2009). Osteoporosis, a generalized decrease in bone mass to at least 2.5 standard deviations below that of an average young adult, results in a lifetime risk of fracture of one in five among men over fifty and one in three among women over fifty (De castro machado, Hannon & Eastell, 1999; Yelin, Weinstein & King, 2016; Sebbag et al., 2019). The high rate of osteoporosis resulting in both direct medical costs and loss of productivity and quality of life is an impetus for improving understanding of additional risk factors and identifying new therapeutic targets.

The disproportionate osteoporosis risk among women is explained mostly by the regulatory role of estrogens in osteoclast differentiation and function. Estradiol limits resorption by both reducing differentiation and limiting the lifespan of osteoclasts (Shevde et al., 2000; Krum et al., 2008). Prior to menopause, ovary-derived estradiol protects young female bone mass. After menopause, which is marked by cessation of ovarian follicle development and a precipitous decline in circulating estradiol, osteoclasts can become disinhibited. If the hyperactivity of postmenopausal osteoclasts cannot be overcome by increased bone-forming activity of osteoblasts, progressive bone loss and osteoporosis follows (Recker et al., 2004; Iki et al., 2004).

Men experience osteoporosis risk differently. Although circulating estradiol level in men of all ages is comparable to that of post-menopausal women, aromatase enzyme (encoded by the CYP19A1 gene) present in the bone converts testosterone to estradiol locally, where it exerts its protective effect (Sasano et al., 1997; Gennari, Nuti & Bilezikian, 2004; Khosla et al., 2005a, 2005b). In contrast to women and estradiol, men do not experience a rapid decrease in testosterone at an age-dependent milestone like menopause, but rather demonstrate increasing variation beginning in the second decade of life (Kelsey et al., 2014). Sex-specific endocrine differences likely account for much of sex-dependent osteoporosis risk, with bone parameters of men in their eighth decade approaching parity with women in their sixth (Alswat, 2017).

While estrogen deficiency explains much of the sex-differential osteoporosis risk, the regulators of osteoclast differentiation and activity are diverse, and there are likely sexually divergent contributors independent of sex steroids. Osteoclasts differentiate from macrophage-like precursors *via* stimulation with Receptor Activator of Nuclear Factor κB Ligand (RANKL). Upon binding of RANKL to its cognate receptor, RANK, Tumor necrosis factor Receptor Associated Factor (TRAF)-dependent signaling cascades converge on multiple transcription factors including JNK, ERK, p38, and NFκB (Feng, 2005). This receptor-proximal signaling alters expression of multiple genes; chief among them is upregulation of Nuclear Factor of Activated T Cells 1 (NFATc1), which drives much of the osteoclast differentiation program (Kim & Kim, 2014, p. 1). Expression of NFATc1 and other osteoclastic genes is also subject to epigenetic regulation (Rohatgi et al., 2018; Das et al., 2018; Shin et al., 2019; Astleford et al., 2020). The complexity of osteoclastic gene expression presents opportunities for variation in degree of differentiation and level of resultant resorptive activity, and multiple pathways other than RANKL/RANK can modulate osteoclastogenesis (Liu et al., 2009; Goel et al., 2019). From this we hypothesized that there are sexually divergent patterns of gene regulation in the differentiation of osteoclasts.

Here we report the findings of our analyses of gene expression and chromatin accessibility in bone marrow macrophages and osteoclasts extracted from female and male mice.

## Materials and Methods

### Osteoclast precursor culture

Mice used as cell sources were euthanized and provided by the staff of the Eastern Washington University vivarium (PHS animal welfare assurance number D19-01059). Primary mouse bone marrow cells were obtained by flushing α-MEM medium (Minimum Essential Medium Eagle (M0894; MilliporeSigma, St. Louis, MO, USA), 20 mM GlutaMAX (35050061; Thermo Fisher, Waltham, MA, USA), and 10% heat-inactivated fetal bovine serum (F-500-D; Atlas Biologicals, Fort Collins, CO, USA)) through the femurs and tibias of 3-month-old C57Bl6 female and male mice (three each for RNA-sequencing; two each for ATAC-Sequencing) (Kaur et al., 2017). Bone marrow cells were cultured at 37 °C and 5% CO2 overnight in tissue culture-treated dishes to allow adherent cells to attach. The next day, non-adherent cells were transferred to non-treated suspension culture dishes and supplemented with 25 ng/mL Macrophage Colony-Stimulating Factor/MCSF (200-08; Shenandoah Biotechnology, Warminster, PA, USA) to induce the differentiation of macrophages, which can attach even to non-treated culture surfaces. After 24 h, the MCSF-containing medium was refreshed to remove non-macrophage cells, and macrophages were maintained for an additional 48 h to allow them to proliferate prior to experimental treatments.

Osteoclasts were TRAP stained according to the University of Rochester Center for Musculoskeletal Research protocol (“Forms and Protocols—Histology Core—Core Services—Center for Musculoskeletal Research—University of Rochester Medical Center”, https://www.urmc.rochester.edu/musculoskeletal-research/core-services/histology/protocols.aspx). Cells were fixed in a solution of 8% neutral buffered formalin (10%, 10790-714; VWR, Radnor, PA, USA), 26% citrate solution (915-50ML, Millipore-Sigma, St. Louis, MO, USA), and 66% acetone (534064-500ML, Millipore-Sigma, St. Louis, MO, USA) for 2 min and washed twice with phosphate buffered saline before a 1 h incubation at 37 °C in a pH 5.0 aqueous solution of 9.2 g/L sodium acetate (S-2889; MilliporeSigma, St. Louis, MO, USA), 11.4g/L L-(+) tartaric acid (T-6521, MilliporeSigma, St. Louis, MO, USA), 2.8 mL/L glacial acetic acid (AX0074-6; MilliporeSigma, St. Louis, MO, USA), 100 mg/L Napthol AS-MX Phosphate (N-4875; MilliporeSigma, St. Louis, MO, USA; freshly dissolved in 2-ethoxyethanol (E-2632; MilliporeSigma, St. Louis, MO, USA) at a concentration of 20 mg/mL), and 600 mg/L Fast Red Violet LB Salt (F-3381; MilliporeSigma, St. Louis, MO, USA). After staining, wells were washed twice with deionized water and allowed to air dry.

### RNA-Sequencing (RNA-Seq)

Appropriate sample sizes were calculated using RNASeqPower with the following parameters: average read depth = 20 million, biological coefficient of variation = 0.1, effect size = 2, alpha = 0.05, power = 0.9 (Hart et al., 2013; Therneau & Stephen, 2022). From this, a sample size of three measurements per group were determined as sufficient. Osteoclast precursors from male and female mice were divided into two treatment groups: one group of cells (macrophages) were maintained in α -MEM with 25 ng/mL MCSF for 72 h; the other group of cells (osteoclasts), were maintained in α -MEM with 25 ng/mL MCSF (200-08; Shenandoah Biotechnology, Warminster, PA, USA) and 100 ng/mL RANKL (200-04; Shenandoah Biotechnology, Warminster, PA, USA) for 72 h. Media were refreshed at 48 h. At the conclusion of the culture period, cells were lysed in TRI reagent and RNA was extracted using the Direct-zol RNA miniprep kit (R2051; Zymo Research, Irvine, CA, USA). RNA was extracted from each group (female-macrophages, female-osteoclasts, male-macrophages, and male-osteoclasts) in triplicate. RNA samples were transferred to GeneWiz/Azenta for cDNA synthesis, indexing, sequencing, and basic RNA-seq analysis (de-multiplexing, alignment, transcript identification, and differential expression analysis). Raw sequence reads were trimmed to remove adaptors and poor sequence quality nucleotides using Trimmomatic v.0.36, trimmed reads were mapped to the GRCm38 *Mus musculus* reference genome using STAR aligner v.2.5.2b, unique gene hit counts from reads falling within exon regions were calculated with the featureCounts module of Subread package v.1.5.2, and differential expressions were calculated using DESeq2 with *p*-values and log2 fold changes determined *via* the Wald test. Differential expression data is included as Supplemental Files. RNA sequencing data is available at NCBI GEO, accession number GSE216929

### Assay for transposase-accessible chromatin-sequencing

Osteoclast precursors from male and female mice were divided into two groups: one group of cells (MCSF only) were maintained in α-MEM with 25 ng/mL MCSF for 24 h; the other group of cells (MCSF+RANKL),were maintained in α-MEM with 25 ng/mL MCSF and 100 ng/mL RANKL for 24 h. At the conclusion of the culture period, nuclei were isolated and used in tagmentation and indexing reactions using the ATAC-Seq Kit (53150, Active Motif, Carlsbad, CA, USA). ATAC-seq libraries were prepared from each sex and treatment group (female-MCSF, female-MCSF+RANKL, male-MCSF, male-MCSF+RANKL) in duplicate. ATAC-seq libraries were transferred to GeneWiz/Azenta, which performed the sequencing reactions and basic ATAC-seq analysis (de-multiplexing, alignment, peak calling, and peak differential analysis). Sequencing adaptors and low-quality nucleotides were removed with Trimmomatic v.0.38, and reads were aligned to the *Mus musculus* mm10 reference genome using bowtie2. Aligned reads were filtered with samtools v1.9 to preserve alignments with a minimum mapping quality of 30, are aligned concordantly, and are the primary called alignments; PCR or digital duplicates were marked with Picard v2.18.26 and removed. Mitochondrial reads and reads mapped to unplaced contigs were also removed. Open chromatin regions were identified through peak calling with MACS2 v2.1.2, and consensus peaks from sample duplicates were merged and kept for downstream analysis. After reads falling beneath peaks were counted, differential peak calling (corresponding to differentially accessible chromatin regions) was performed using the R package Diffbind. Differential peak call data are provided as Supplemental Files.

### Pathway analyses of differential sequencing data

Differential data with non-adjusted *p*-values were uploaded to iPathwayGuide (Advaita Bioinformatics; http://www.advaitabio.com/ipathwayguide). iPathwayGuide is a web-based bioinformatics platform that applies Impact Analysis to differential datasets, which considers both over-representation of differential genes in a particular pathway and computed perturbation to generate pathway specific *p*-values (Tarca et al., 2009; Ahsan & Drăghici, 2017). *p*-Values for individual genes were adjusted *via* False Discovery Rate. Venn diagrams were generated with eulerr.co (Larsson, Godfrey & Gustafsson, 2021).

## Results

### Gene expression patterns in macrophages and osteoclasts cluster by sex and differentiation state

**Figure 1:**
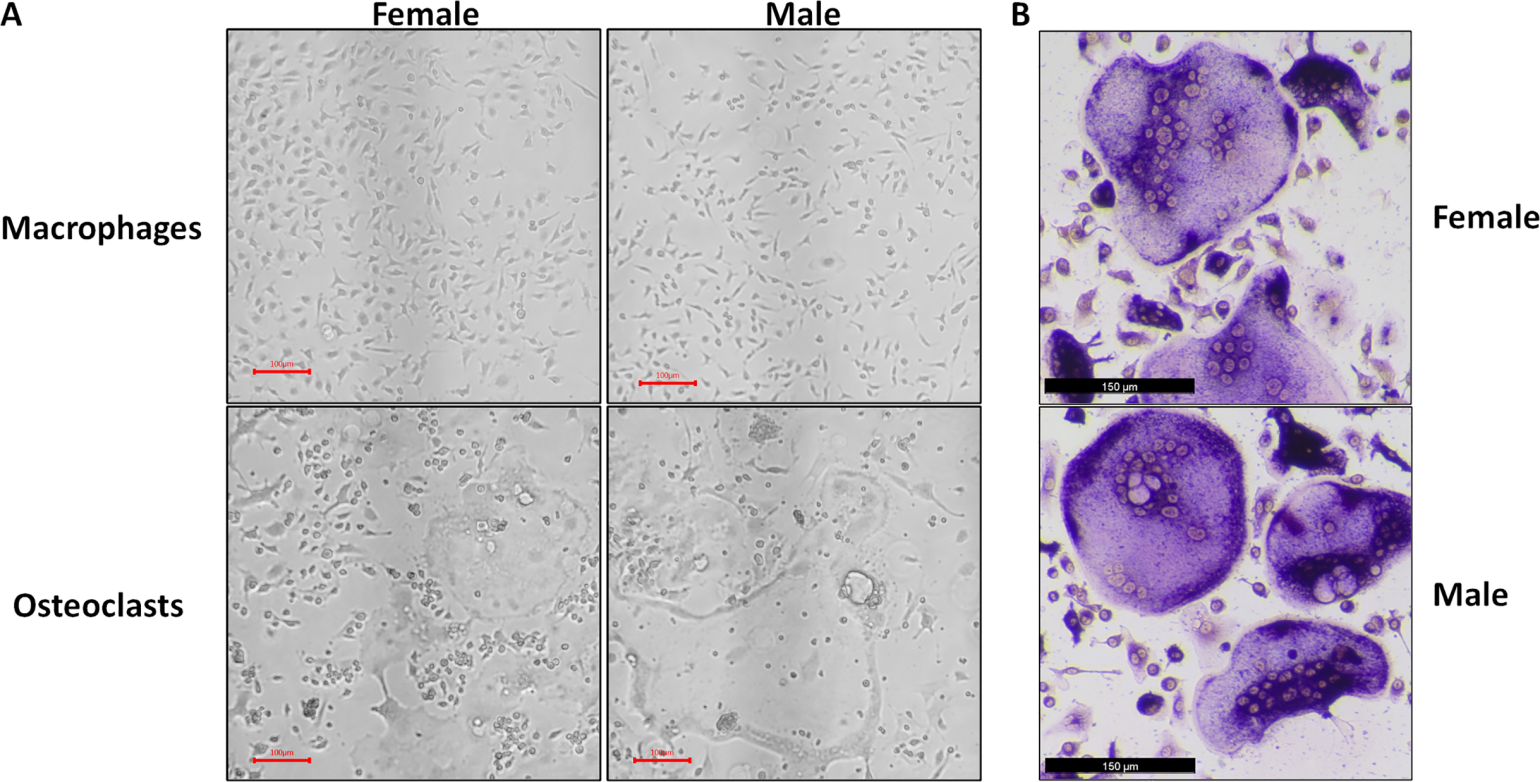
Representative micrographs of undifferentiated macrophages and osteoclasts. Male and female bone marrow-derived macrophages were cultured with 25 ng/mL MCSF alone or 25 ng/mL MCSF and 100 ng/mL RANKL for 3 days. (A) Micrographs of macrophages and osteoclasts prior to lysis and RNA extraction. Cells cultured with RANKL demonstrated multinucleation and increased size. (B) Micrographs of TRAP-stained osteoclast differentiated under conditions matched to RNA source cells.

**Figure 2:**
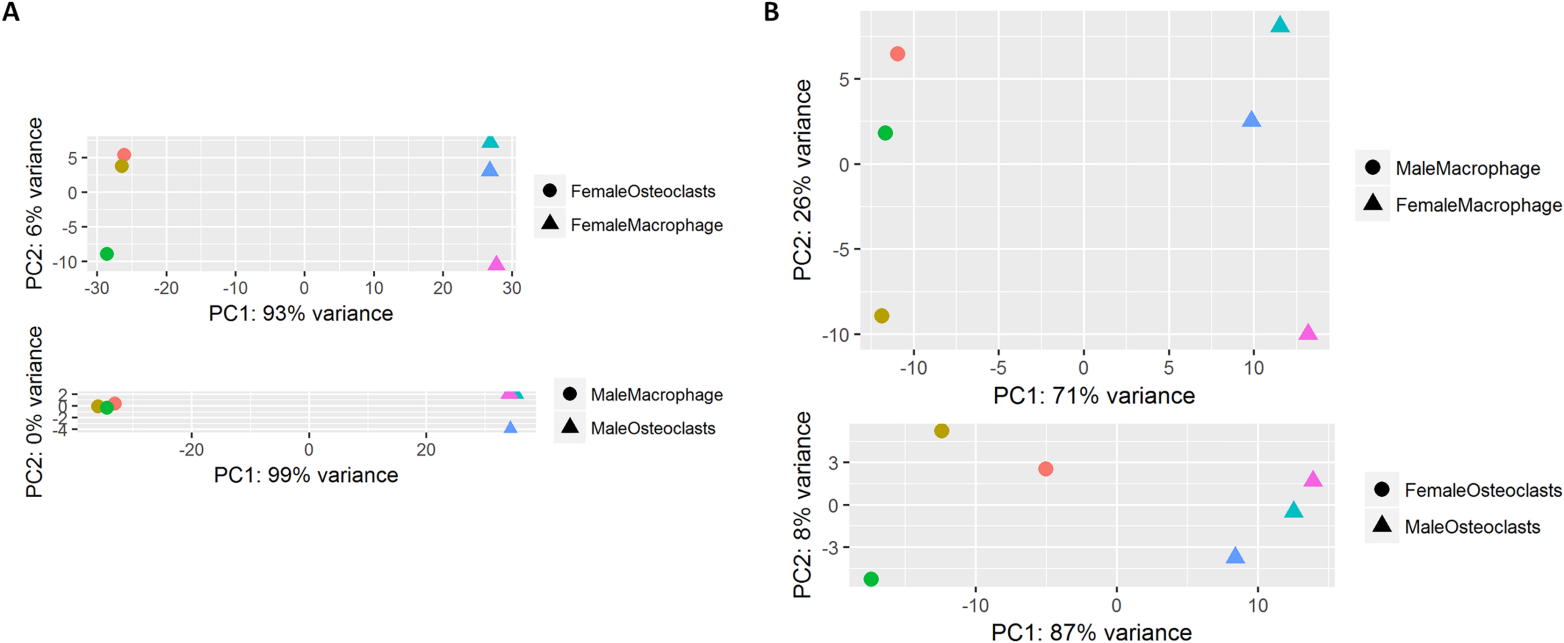
Principal component analysis of RNA-Seq data. (A) RNA-seq replicates from male and female samples demonstrate PC1 clustering according to differentiation state. (B) RNA-seq replicates from macrophages and osteoclasts demonstrate PC1 clustering according to sex.

At the conclusion of the treatment period, cells cultured with MCSF alone retained the typical fusiform morphology of immunologically naïve macrophages, where MCSF and RANKL treatment resulted in large, multinucleated osteoclasts (Fig. 1A). Osteoclasts differentiated under matched conditions were TRAP positive (Fig. 1B). Following RNA-seq, principal component analysis of replicates from each group revealed that in both sexes, the principal component of clustering (PC1) was differentiation state (Fig. 2A). Similarly, when comparing cells of the same differentiation state, sex of the cells was the principal component (Fig. 2B).

### RNA-sequencing of macrophages and osteoclasts identifies expected differential gene expression

**Figure 3:**
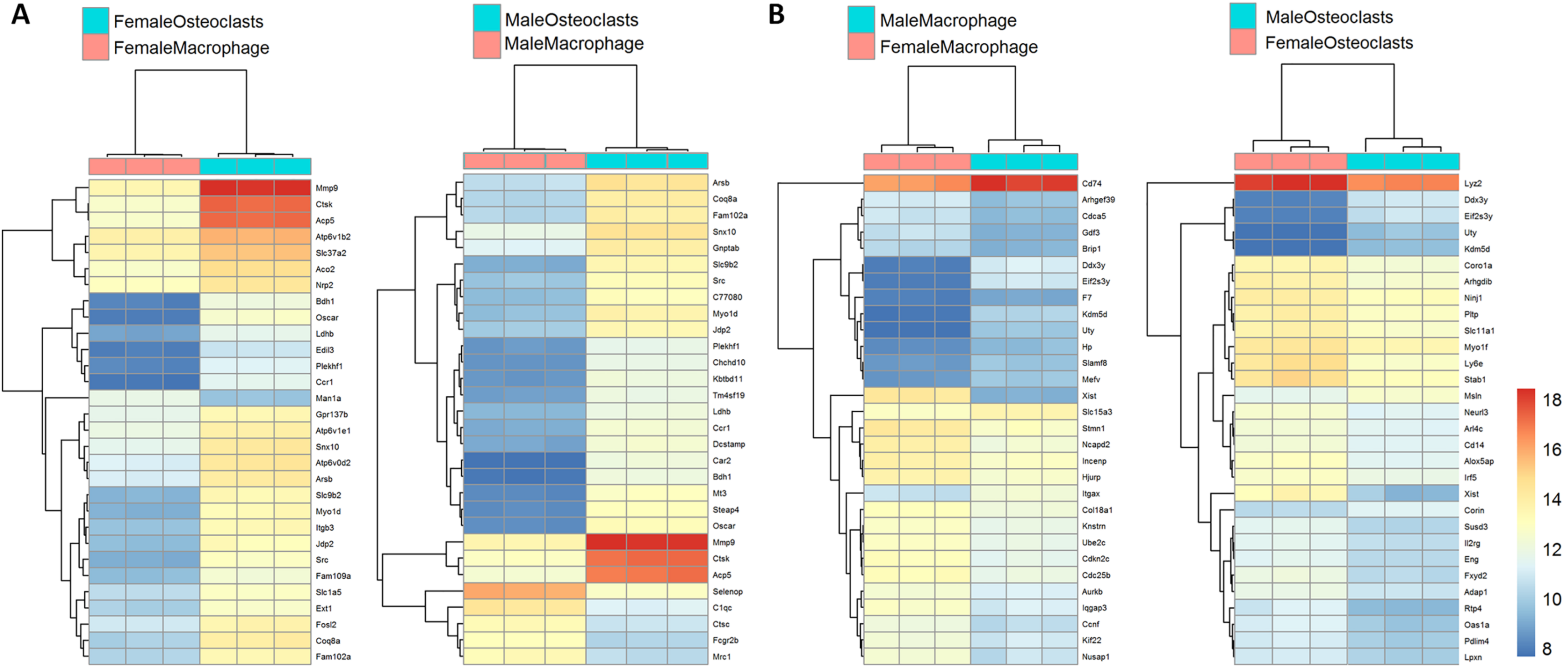
Biclustering analyses of top 30 differentially expressed genes. (A) Differentially regulated genes in male and female cells by differentiation state. In both male and female cells, osteoclastic genes MMP9, Ctsk, and Acp5 demonstrate the most significant differences in expression. (B) Differentially regulated genes in macrophages and osteoclasts by sex. Expected female (XIST) and male (Ddx3y, Uty, and Eif2s3y) differential genes were identified in both cell types.

Figure 3 depicts biclustering analyses of 30 differentially expressed genes with the lowest adjusted *p*-values according to differentiation state and sex. Among both male and female cells, matrix metalloproteinase 9 (Mmp9), cathepsin K (Ctsk), and tartrate-resistant acid phosphatase (Acp5), which are involved in osteoclast function, were among the most significantly differential (Fig. 3A). When comparing cells of the same differentiation state and opposite sexes, Ubiquitously Transcribed Tetratricopeptide Repeat Containing, Y-Linked (Uty), DEAD-Box Helicase 3 Y-Linked (Ddx3y), and Eukaryotic translation initiation factor 2 subunit 3, Y-linked (Eif2s3y), which are restricted to the Y chromosome, were found to be more highly expressed in male cells. Conversely, X-inactive specific transcript (Xist), which is expressed only in cells with more than one X chromosome, was more highly expressed in female cells (Fig. 3B). The identification of expected differences in gene expression by the unbiased RNA-seq method increases confidence in the method and subsequent analyses to identify novel differential gene expression based on sex and differentiation state.

### Sex-independent and sex-dependent differential pathways in osteoclastogenesis

**Figure 4:**
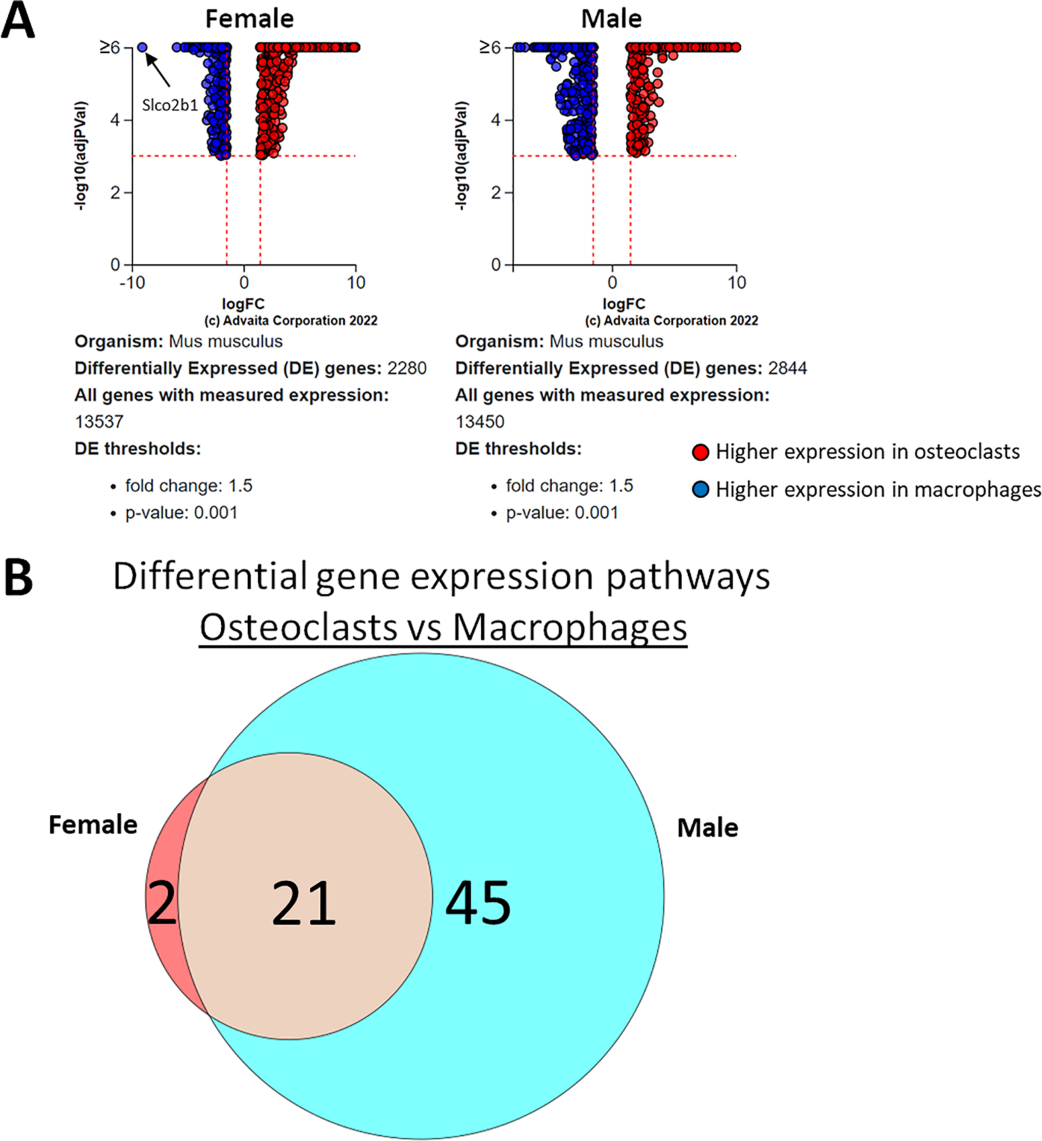
Gene and pathway analysis of osteoclastogenesis RNA-Seq data. (A) Volcano plots depicting genes meeting fold change and *p*-value thresholds in female and male cells. Differentials with −log10 transformed adjusted *p*-values of six and greater are plotted at the same level. Full volcano plots are included in Fig. S1. (B) Female-specific, male-specific, and common pathways altered during osteoclastogenesis. Pathways are listed in Table 1.

Using a *p*-value threshold of 0.001 and a minimum fold change of 1.5, 2,280 differentially expressed genes were identified in female cells and 2,844 in male (Fig. 4A). Following false discovery rate correction, pathway analysis of differential gene expression according to differentiation state identified 22 altered pathways common to both female and male cells, 45 specific to male cells, and two specific to female cells (Fig. 4B). These pathways are listed in Table 1.

**Table 1:** Altered transcriptional pathways during osteoclastogenesis.

Female-specific		Male-specific		Sex-independent		
Pathway	*p*-value	Pathway	*p*-value	Pathway	*p*-value (Female)	*p*-value (Male)
Amyotrophic lateral sclerosis	9.91E−05	Cytokine-cytokine receptor interaction	1.82E−06	Non-alcoholic fatty liver disease	8.23E−05	5.48E−05
Taste transduction	0.016	Viral protein interaction with cytokine and cytokine receptor	1.82E−06	Huntington disease	9.91E−05	0.009
		Antigen processing and presentation	1.28E−05	Thermogenesis	1.98E−04	3.63E−04
		*Staphylococcus aureus* infection	1.73E−05	Retrograde endocannabinoid signaling	1.98E−04	0.007
		Influenza A	2.03E−05	Cardiac muscle contraction	2.06E−04	9.33E−05
		Allograft rejection	5.09E−05	Citrate cycle (TCA cycle)	2.06E−04	9.33E−05
		Graft-*versus*-host disease	5.09E−05	Collecting duct acid secretion	2.06E−04	9.33E−05
		Intestinal immune network for IgA production	5.09E−05	Metabolic pathways	2.06E−04	9.33E−05
		Toll-like receptor signaling pathway	5.09E−05	Oxidative phosphorylation	2.06E−04	9.33E−05
		Type I diabetes mellitus	5.09E−05	Parkinson disease	2.06E−04	0.006
		Autoimmune thyroid disease	6.40E−05	Pathways of neurodegeneration-multiple diseases	2.06E−04	0.046
		Inflammatory bowel disease	7.05E−05	Prion disease	2.68E−04	6.78E−05
		Chemokine signaling pathway	8.80E−05	Alzheimer disease	2.68E−04	0.009
		Hematopoietic cell lineage	9.33E−05	Synaptic vesicle cycle	7.17E−04	3.63E−04
		Viral myocarditis	9.33E−05	Phagosome	0.001	9.33E−05
		Malaria	9.46E−05	Neuroactive ligand-receptor interaction	0.002	5.09E−05
		Tuberculosis	1.38E−04	Rheumatoid arthritis	0.002	8.80E−05
		Asthma	1.52E−04	Calcium signaling pathway	0.002	3.09E−04
		Measles	3.63E−04	Axon guidance	0.003	0.004
		NOD-like receptor signaling pathway	4.40E−04	Cell adhesion molecules	0.005	9.33E−05
		Bile secretion	0.002	Systemic lupus erythematosus	0.022	1.87E−05
		Pertussis	0.002			
		Osteoclast differentiation	0.003			
		Hepatitis B	0.004			
		Human cytomegalovirus infection	0.005			
		Legionellosis	0.007			
		Pathways in cancer	0.007			
		Arachidonic acid metabolism	0.009			
		cAMP signaling pathway	0.009			
		Toxoplasmosis	0.009			
		Lysosome	0.011			
		Th17 cell differentiation	0.011			
		Th1 and Th2 cell differentiation	0.012			
		MAPK signaling pathway	0.013			
		C-type lectin receptor signaling pathway	0.014			
		Pyruvate metabolism	0.018			
		Necroptosis	0.02			
		Human papillomavirus infection	0.021			
		ABC transporters	0.024			
		Epstein-Barr virus infection	0.035			
		Herpes simplex virus 1 infection	0.042			
		Rap1 signaling pathway	0.043			
		cGMP-PKG signaling pathway	0.044			
		NF-kappa B signaling pathway	0.045			
		TNF signaling pathway	0.046			

### Differentiation-independent and differentiation-dependent differential pathways in female and male cells

**Figure 5:**
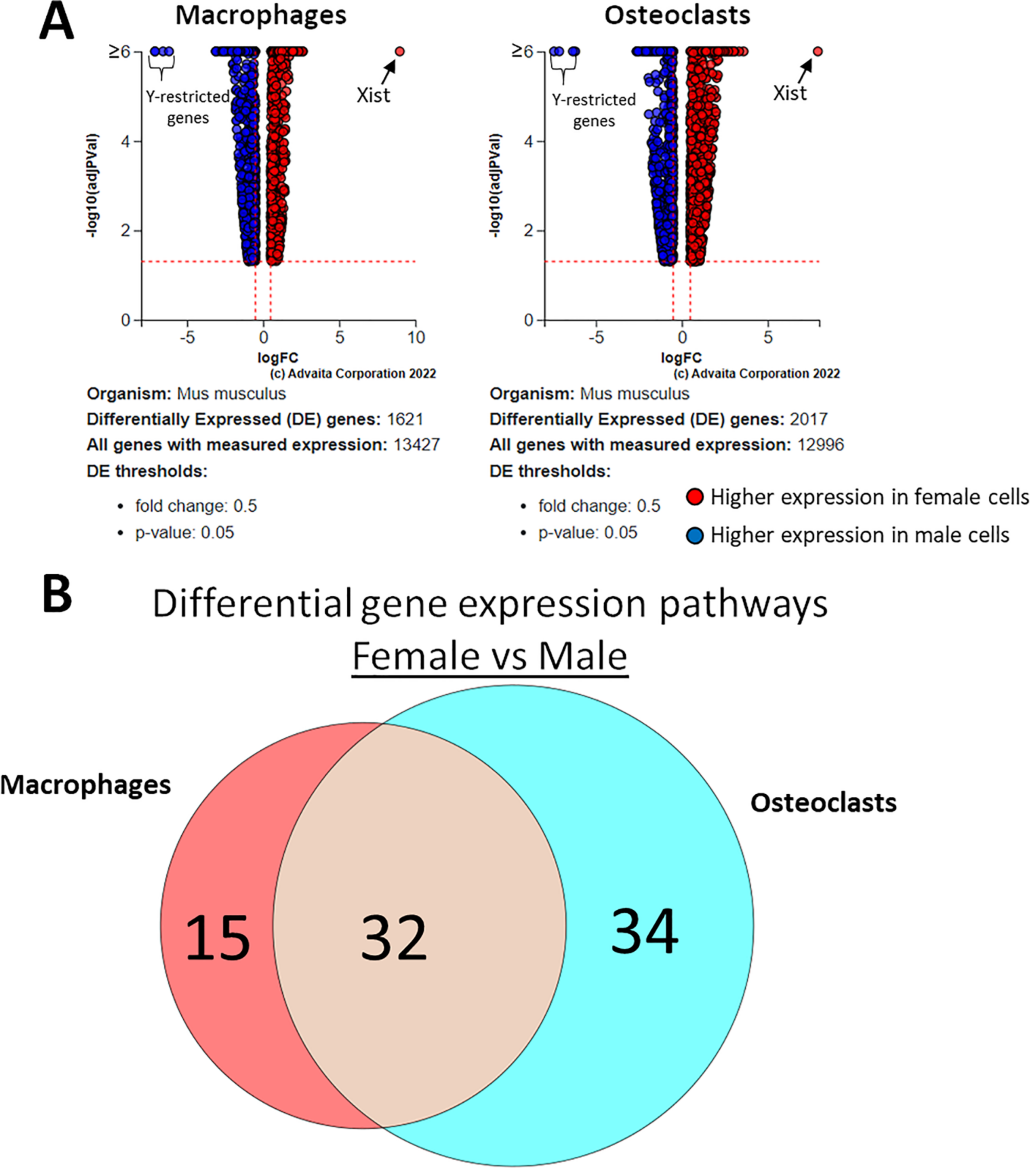
Gene and pathway analysis of sexually divergent RNA-Seq data. (A) Volcano plots depicting genes meeting fold change and *p*-value thresholds in macrophages and osteoclasts. Differentials with −log10 transformed adjusted *p*-values of 6 and greater are plotted at the same level. Full volcano plots are included in Fig. S1. (B) Macrophage-specific, osteoclast-specific, and common pathways altered during osteoclastogenesis. Pathways are listed in Table 2.

Using a *p*-value threshold of 0.05 and a minimum fold change of 0.6, 1,347 differentially expressed genes were identified in macrophages and 1,618 in osteoclasts (Fig. 5A). Following false discovery rate correction, pathway analysis of differential gene expression according to sex identified 32 altered pathways common to both macrophages and osteoclasts, 15 specific to macrophages, and 34 specific to osteoclasts (Fig. 5B). These pathways are listed in Table 2.

**Table 2:** Altered transcriptional pathways between sexes.

Macrophage-specific		Osteoclast-specific		Differentiation-independent		
Pathway	*p*-value	Pathway	*p*-value	Pathway	*p*-value (macrophage)	*p*-value (osteoclast)
Fanconi anemia pathway	2.09E−05	Measles	8.40E−05	Cytokine-cytokine receptor interaction	9.46E−06	5.16E−05
Cell cycle	7.15E−05	Inflammatory bowel disease	2.23E−04	Viral protein interaction with cytokine and cytokine receptor	2.09E−05	1.24E−04
Asthma	2.19E−04	Pertussis	2.23E−04	*Staphylococcus aureus* infection	2.19E−04	2.49E−05
DNA replication	2.19E−04	Kaposi sarcoma-associated herpesvirus infection	2.44E−04	Cell adhesion molecules	2.19E−04	2.02E−04
Homologous recombination	2.19E−04	Axon guidance	6.32E−04	Systemic lupus erythematosus	2.19E−04	2.44E−04
IL-17 signaling pathway	0.002	Human cytomegalovirus infection	6.32E−04	Hematopoietic cell lineage	2.19E−04	2.82E−04
Progesterone-mediated oocyte maturation	0.011	Pathways in cancer	0.001	Leishmaniasis	2.19E−04	0.002
Fluid shear stress and atherosclerosis	0.015	MAPK signaling pathway	0.002	Rheumatoid arthritis	2.41E−04	5.52E−04
Cellular senescence	0.021	ABC transporters	0.003	Malaria	3.04E−04	1.88E−04
Oocyte meiosis	0.022	Hepatitis B	0.003	Autoimmune thyroid disease	0.001	2.23E−04
Mismatch repair	0.029	Cytosolic DNA-sensing pathway	0.004	C-type lectin receptor signaling pathway	0.001	0.01
NF-kappa B signaling pathway	0.031	Necroptosis	0.004	Tuberculosis	0.002	2.01E−04
ECM-receptor interaction	0.041	Hepatitis C	0.005	Allograft rejection	0.002	2.23E−04
p53 signaling pathway	0.048	Bile secretion	0.007	Antigen processing and presentation	0.004	1.31E−05
Small cell lung cancer	0.049	Hypertrophic cardiomyopathy	0.007	Type I diabetes mellitus	0.004	2.23E−04
		Dilated cardiomyopathy	0.008	Graft-*versus*-host disease	0.004	2.23E−04
		Mineral absorption	0.008	Chemokine signaling pathway	0.004	8.82E−04
		cGMP-PKG signaling pathway	0.009	Intestinal immune network for IgA production	0.006	0.007
		Chagas disease	0.009	Complement and coagulation cascades	0.008	0.026
		Legionellosis	0.016	Influenza A	0.01	1.83E−05
		Herpes simplex virus 1 infection	0.017	Viral myocarditis	0.01	8.40E−05
		Human papillomavirus infection	0.017	Osteoclast differentiation	0.01	0.001
		PI3K-Akt signaling pathway	0.023	NOD-like receptor signaling pathway	0.015	2.23E−04
		AGE-RAGE signaling pathway in diabetic complications	0.027	Toll-like receptor signaling pathway	0.021	1.32E−04
		GABAergic synapse	0.027	Transcriptional misregulation in cancer	0.021	0.012
		Oxytocin signaling pathway	0.027	TNF signaling pathway	0.021	0.025
		Arachidonic acid metabolism	0.03	Toxoplasmosis	0.022	0.014
		Salmonella infection	0.032	Neuroactive ligand-receptor interaction	0.028	1.88E−04
		Human immunodeficiency virus 1 infection	0.038	Human T-cell leukemia virus 1 infection	0.028	0.002
		Rap1 signaling pathway	0.038	Epstein-Barr virus infection	0.031	4.31E−04
		Circadian entrainment	0.04	Phagosome	0.041	6.41E−04
		Protein digestion and absorption	0.04	Th17 cell differentiation	0.041	0.019
		Salivary secretion	0.04			
		Adrenergic signaling in cardiomyocytes	0.042			

### Differential transposase accessible chromatin regions regulated by RANKL in female and male cells

**Figure 6:**
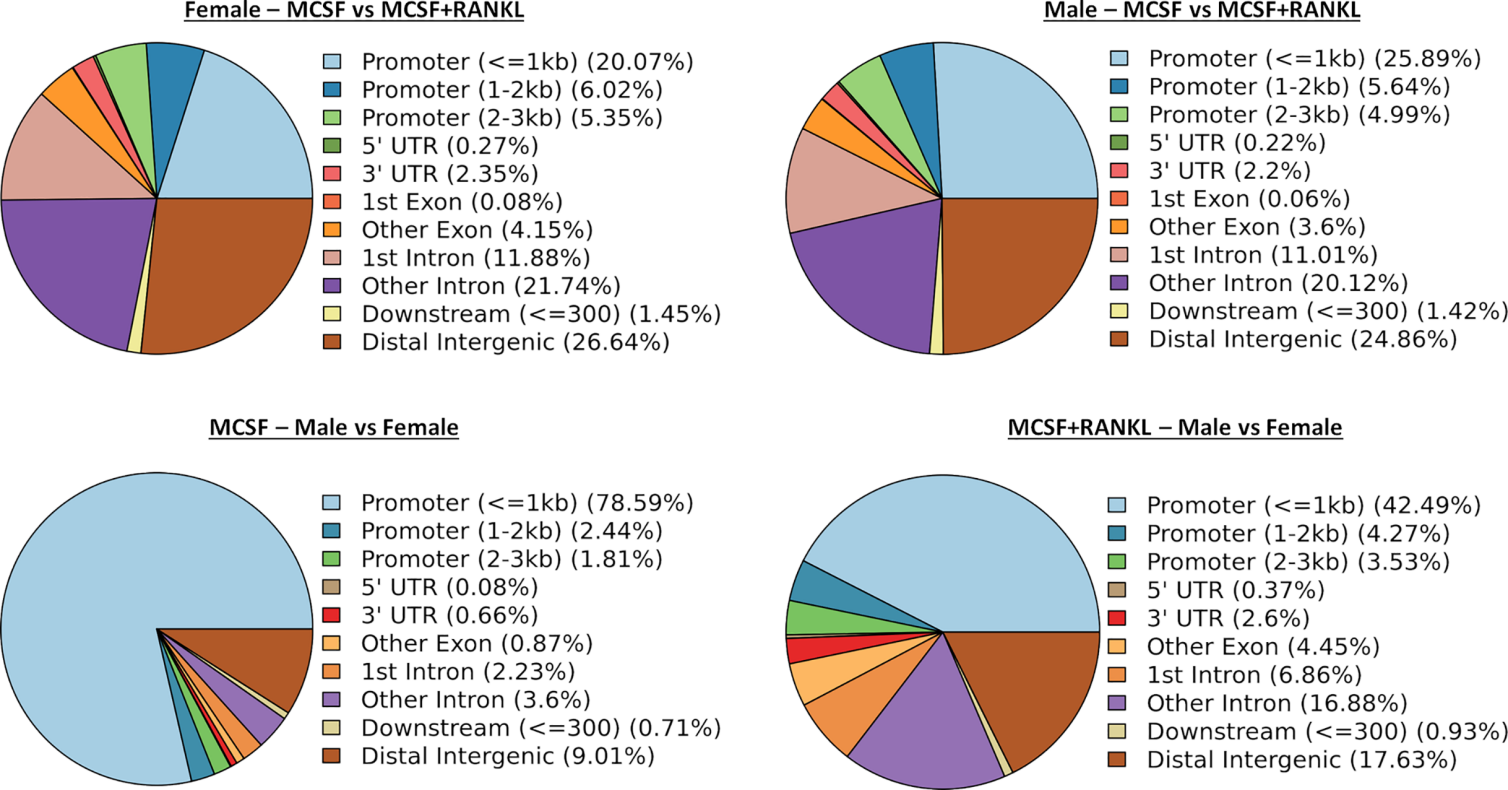
Differentially accessible chromosome locations in female and male macrophages cultured with MCSF alone or MCSF and RANKL. Most differential accessible regions were located within promoter regions.

**Figure 7:**
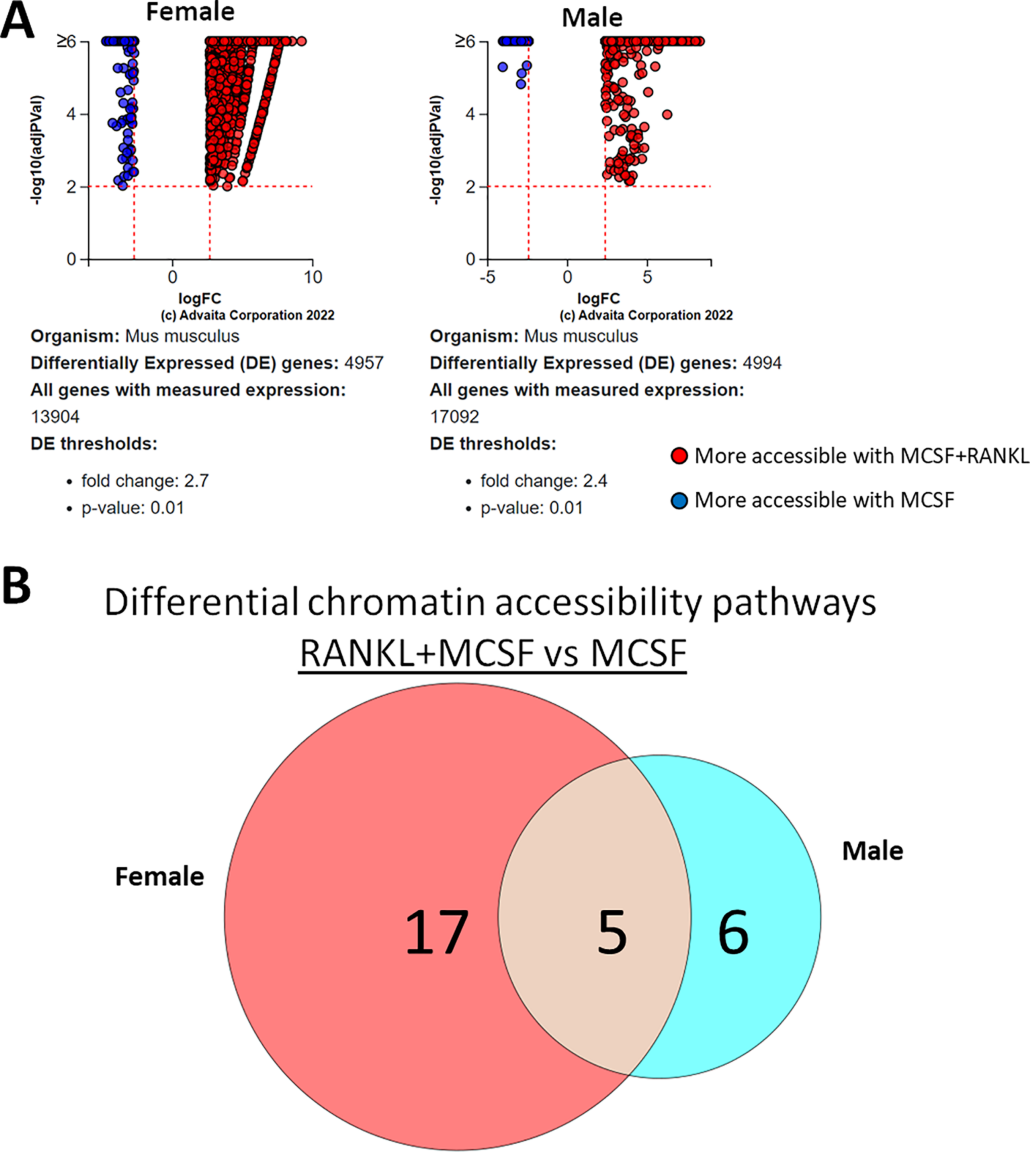
Gene and pathway analysis of osteoclastogenesis ATAC-Seq data. (A) Volcano plots depicting genes meeting fold change and *p*-value thresholds in female and male cells. Differentials with −log10 transformed adjusted *p*-values of six and greater are plotted at the same level. Full volcano plots are included in Fig. S1. (B) Female-specific, male-specific, and common pathways altered during osteoclastogenesis. Pathways are listed in Table 3.

ATAC-seq revealed that most differences in transposase-accessible chromatin were in promotor and distal intergenic regions (Fig. 6). Using a *p*-value threshold of 0.01 and a minimum accessibility fold difference of 2.7 (female) or 2.4 (male), there were 4,957 genes with differentially accessible chromatin in female MCSF+RANKL-treated cells and 4,994 in male (Fig. 7A). Most differentially accessible genes were more accessible following MCSF+RANKL treatment with more accessible genes accounting for 95% in female cells and 94% in male cells. Without FDR correction, there were 17 differentially accessible pathways specific to female cells, six specific to male cells, and five common to both sexes (Fig. 7B). These pathways are listed in Table 3.

**Table 3:** Differential chromatin accessible pathways during osteoclastogenesis.

Female-specific		Male-specific		Sex-independent		
Pathway	*p*-value	Pathway	*p*-value	Pathway	*p*-value (Female)	*p*-value (Male)
Small cell lung cancer	0.006	Protein digestion and absorption	0.008	Hippo signaling pathway	7.52E−04	0.004
Endocrine resistance	0.007	cGMP-PKG signaling pathway	0.012	Axon guidance	8.12E−04	0.005
Prion disease	0.009	Pancreatic secretion	0.018	Focal adhesion	0.001	0.007
Alpha-Linolenic acid metabolism	0.012	Arrhythmogenic right ventricular cardiomyopathy	0.019	ECM-receptor interaction	0.002	0.004
Growth hormone synthesis, secretion and action	0.015	Ubiquinone and other terpenoid-quinone biosynthesis	0.031	Insulin secretion	0.035	0.04
Platinum drug resistance	0.018	Aldosterone synthesis and secretion	0.043			
HIF-1 signaling pathway	0.025					
Relaxin signaling pathway	0.025					
Rap1 signaling pathway	0.029					
Fluid shear stress and atherosclerosis	0.041					
Glycosaminoglycan biosynthesis—heparan sulfate/heparin	0.041					
Linoleic acid metabolism	0.041					
Central carbon metabolism in cancer	0.042					
Estrogen signaling pathway	0.042					
PI3K-Akt signaling pathway	0.044					
Proteoglycans in cancer	0.045					
Necroptosis	0.048					

### Sex-independent and sex-dependent differential pathways in differential transposase accessible chromatin regions in MCSF- and MCSF+RANKL-treated cells

**Figure 8:**
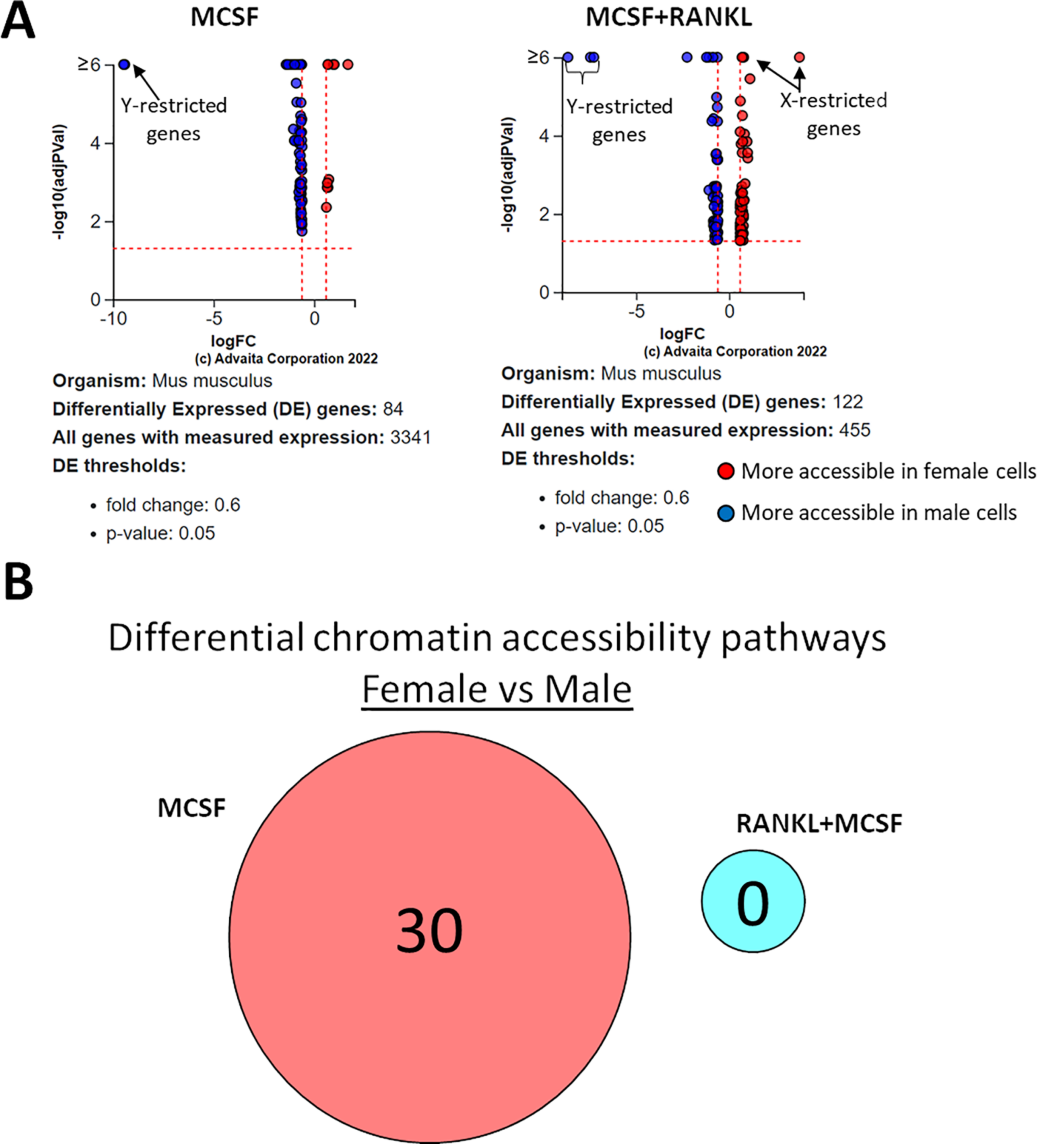
Gene and pathway analysis of sexually divergent ATAC-Seq data. (A) Volcano plots depicting genes meeting fold change and *p*-value thresholds in MCSF and MCSF+RANKL treated cells. Differentials with −log10 transformed adjusted *p*-values of six and greater are plotted at the same level. Full volcano plots are included in Fig. S1. (B) MCSF-specific, MCSF+RANKL-specific, and common pathways altered during osteoclastogenesis. Pathways are listed in Table 4.

Using a *p*-value threshold of 0.05 and a minimum accessibility fold difference of 0.6, there were 84 genes with differentially accessible chromatin in MCSF-treated cells and 122 in MCSF+RANKL-treated cells (Fig. 8A). Without FDR correction, there were 30 differentially accessible pathways in MCSF-treated cells (Fig. 8B). These pathways are listed in Table 4.

**Table 4:** Differential chromatin accessible pathways between sexes.

MCSF-specific		MCSF+RANKL specific	Treatment-independent
Pathway	*p*-value	Pathway	Pathway
Influenza A	2.76E−05	NONE	NONE
Antigen processing and presentation	4.67E−05		
Viral myocarditis	5.94E−05		
Th17 cell differentiation	6.01E−05		
Asthma	7.99E−05		
Leishmaniasis	1.72E−04		
*Staphylococcus aureus* infection	2.97E−04		
Allograft rejection	3.92E−04		
Autoimmune thyroid disease	3.92E−04		
Graft-*versus*-host disease	3.92E−04		
Type I diabetes mellitus	3.92E−04		
Th1 and Th2 cell differentiation	4.77E−04		
Inflammatory bowel disease	6.85E−04		
Rheumatoid arthritis	7.02E−04		
Intestinal immune network for IgA production	8.25E−04		
Toxoplasmosis	0.002		
Tuberculosis	0.002		
Cell adhesion molecules	0.003		
Hematopoietic cell lineage	0.003		
Systemic lupus erythematosus	0.003		
TGF-beta signaling pathway	0.004		
Phagosome	0.005		
Transcriptional misregulation in cancer	0.007		
Human T-cell leukemia virus 1 infection	0.011		
IL-17 signaling pathway	0.014		
Rap1 signaling pathway	0.019		
Herpes simplex virus 1 infection	0.031		
Cholesterol metabolism	0.033		
Epstein-Barr virus infection	0.037		
ECM-receptor interaction	0.046		

### Differential gene expression and gene accessibility during osteoclastogenesis

**Figure 9:**
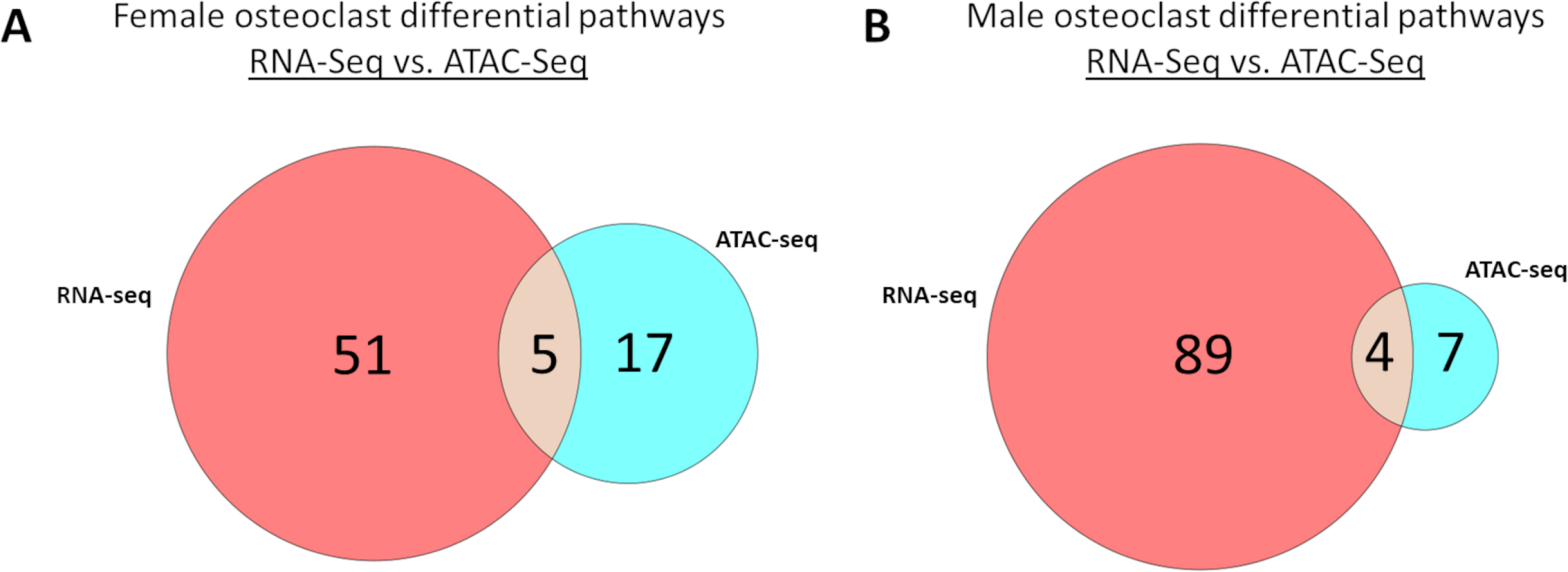
Correlated pathways identified *via* RNA-Seq and ATAC-Seq. (A) RNA-Seq-specific, ATAC-specific, and common pathways in female cells. Pathways are listed in Table 5. (B) RNA-Seq-specific, ATAC-specific, and common pathways in male cells. Pathways are listed in Table 6.

Meta-analysis of macrophage *vs* osteoclast RNA-seq and MCSF *vs* MCSF+RANKL ATAC-seq data revealed 51 gene expression specific pathways, 17 gene accessibility specific pathways, and five pathways common to both in female cells (Fig. 9A). These pathways are listed in Table 5. Among male cells, there were 89 gene expression specific pathways, seven gene accessibility specific pathways, and four common pathways (Fig. 9B). These pathways are listed in Table 6.

**Table 5:** Differential transcriptional and chromatin accessible pathways during osteoclastogenesis in female cells.

RNA-seq only		ATAC-seq only		Common		
Pathway	*p*-value	Pathway	*p*-value	Pathway	*p*-value (RNA-seq)	*p*-value (ATAC-Seq)
Non-alcoholic fatty liver disease	2.58E−07	Hippo signaling pathway	7.52E−04	Prion disease	1.13E−05	0.009
Amyotrophic lateral sclerosis	9.07E−07	Focal adhesion	0.001	Axon guidance	1.96E−04	8.12E−04
Huntington disease	9.32E−07	Small cell lung cancer	0.006	Estrogen signaling pathway	0.013	0.042
Thermogenesis	2.79E−06	Endocrine resistance	0.007	Rap1 signaling pathway	0.026	0.029
Retrograde endocannabinoid signaling	3.10E−06	Alpha-Linolenic acid metabolism	0.012	ECM-receptor interaction	0.028	0.002
Pathways of neurodegeneration-multiple diseases	6.85E−06	Growth hormone synthesis, secretion and action	0.015			
Parkinson disease	6.86E−06	Platinum drug resistance	0.018			
Cardiac muscle contraction	7.75E−06	HIF-1 signaling pathway	0.025			
Citrate cycle (TCA cycle)	7.75E−06	Relaxin signaling pathway	0.025			
Collecting duct acid secretion	7.75E−06	Insulin secretion	0.035			
Metabolic pathways	7.75E−06	Fluid shear stress and atherosclerosis	0.041			
Oxidative phosphorylation	7.75E−06	Glycosaminoglycan biosynthesis—heparan sulfate/heparin	0.041			
Alzheimer disease	1.18E−05	Linoleic acid metabolism	0.041			
Synaptic vesicle cycle	3.37E−05	Central carbon metabolism in cancer	0.042			
Phagosome	6.04E−05	PI3K-Akt signaling pathway	0.044			
Rheumatoid arthritis	9.49E−05	Proteoglycans in cancer	0.045			
Calcium signaling pathway	1.39E−04	Necroptosis	0.048			
Neuroactive ligand-receptor interaction	1.39E−04					
Cell adhesion molecules	3.59E−04					
Taste transduction	0.001					
Systemic lupus erythematosus	0.002					
Arrhythmogenic right ventricular cardiomyopathy	0.004					
cAMP signaling pathway	0.004					
Pyruvate metabolism	0.004					
Regulation of actin cytoskeleton	0.005					
Carbon metabolism	0.006					
*Staphylococcus aureus* infection	0.006					
Complement and coagulation cascades	0.008					
Dilated cardiomyopathy	0.008					
Viral myocarditis	0.008					
Hypertrophic cardiomyopathy	0.013					
Hematopoietic cell lineage	0.017					
Pancreatic secretion	0.017					
Transcriptional misregulation in cancer	0.017					
Cytokine-cytokine receptor interaction	0.021					
Pathways in cancer	0.024					
Chemokine signaling pathway	0.027					
ECM-receptor interaction	0.028					
Arachidonic acid metabolism	0.03					
Gap junction	0.031					
Herpes simplex virus 1 infection	0.031					
GABAergic synapse	0.033					
Glycosaminoglycan degradation	0.033					
Glycosphingolipid biosynthesis - lacto and neolacto series	0.033					
Cellular senescence	0.037					
Glutathione metabolism	0.038					
p53 signaling pathway	0.038					
Gastric acid secretion	0.039					
Glycolysis/Gluconeogenesis	0.047					
Phospholipase D signaling pathway	0.048					
Progesterone-mediated oocyte maturation	0.048					
Cholinergic synapse	0.05					

**Table 6:** Differential transcriptional and chromatin accessible pathways during osteoclastogenesis in male cells.

RNA-seq only		ATAC-seq only		Common		
Pathway	*p*-value	Pathway	*p*-value	Pathway	*p*-value (RNA-Seq)	*p*-value (ATAC-Seq)
Cytokine-cytokine receptor interaction	1.12E−08	ECM-receptor interaction	0.004	Arrhythmogenic right ventricular cardiomyopathy	0.031	0.019
Viral protein interaction with cytokine and cytokine receptor	1.12E−08	Hippo signaling pathway	0.004	Axon guidance	5.41E−04	0.005
Antigen processing and presentation	1.18E−07	Focal adhesion	0.007	cGMP-PKG signaling pathway	0.009	0.012
*Staphylococcus aureus* infection	2.12E−07	Protein digestion and absorption	0.008	Pancreatic secretion	0.013	0.018
Systemic lupus erythematosus	2.87E−07	Ubiquinone and other terpenoid-quinone biosynthesis	0.031			
Influenza A	3.74E−07	Insulin secretion	0.04			
Neuroactive ligand-receptor interaction	1.45E−06	Aldosterone synthesis and secretion	0.043			
Type I diabetes mellitus	1.64E−06					
Toll-like receptor signaling pathway	1.70E−06					
Graft-*versus*-host disease	1.74E−06					
Allograft rejection	1.78E−06					
Intestinal immune network for IgA production	1.88E−06					
Non-alcoholic fatty liver disease	2.19E−06					
Autoimmune thyroid disease	2.76E−06					
Prion disease	3.13E−06					
Inflammatory bowel disease	3.47E−06					
Chemokine signaling pathway	4.75E−06					
Rheumatoid arthritis	4.87E−06					
Viral myocarditis	7.22E−06					
Cardiac muscle contraction	7.75E−06					
Cell adhesion molecules	7.75E−06					
Citrate cycle (TCA cycle)	7.75E−06					
Collecting duct acid secretion	7.75E−06					
Hematopoietic cell lineage	7.75E−06					
Metabolic pathways	7.75E−06					
Oxidative phosphorylation	7.75E−06					
Phagosome	7.75E−06					
Malaria	8.15E−06					
Tuberculosis	1.23E−05					
Asthma	1.41E−05					
Calcium signaling pathway	2.95E−05					
Thermogenesis	3.67E−05					
Measles	3.70E−05					
Synaptic vesicle cycle	3.80E−05					
NOD-like receptor signaling pathway	4.74E−05					
Bile secretion	1.81E−04					
Pertussis	2.52E−04					
Osteoclast differentiation	2.97E−04					
Hepatitis B	5.19E−04					
Human cytomegalovirus infection	6.35E−04					
Parkinson disease	7.15E−04					
Retrograde endocannabinoid signaling	8.66E−04					
Legionellosis	8.87E−04					
Alzheimer disease	0.001					
Arachidonic acid metabolism	0.001					
cAMP signaling pathway	0.001					
Huntington disease	0.001					
Pathways in cancer	0.001					
Toxoplasmosis	0.001					
C-type lectin receptor signaling pathway	0.002					
Lysosome	0.002					
MAPK signaling pathway	0.002					
Th1 and Th2 cell differentiation	0.002					
Th17 cell differentiation	0.002					
Necroptosis	0.003					
Pyruvate metabolism	0.003					
ABC transporters	0.004					
Human papillomavirus infection	0.004					
Epstein-Barr virus infection	0.007					
Herpes simplex virus 1 infection	0.008					
Rap1 signaling pathway	0.008					
NF-kappa B signaling pathway	0.009					
Pathways of neurodegeneration—multiple diseases	0.009					
TNF signaling pathway	0.009					
Complement and coagulation cascades	0.011					
Leishmaniasis	0.016					
Salmonella infection	0.016					
Estrogen signaling pathway	0.017					
Mineral absorption	0.017					
Regulation of actin cytoskeleton	0.021					
Chagas disease	0.022					
JAK-STAT signaling pathway	0.023					
Human immunodeficiency virus 1 infection	0.026					
Salivary secretion	0.026					
Carbon metabolism	0.027					
Dilated cardiomyopathy	0.028					
Gastric acid secretion	0.029					
Taste transduction	0.029					
Proximal tubule bicarbonate reclamation	0.03					
Glycosaminoglycan degradation	0.031					
Transcriptional misregulation in cancer	0.031					
Sphingolipid metabolism	0.032					
Primary bile acid biosynthesis	0.04					
Morphine addiction	0.043					
Inflammatory mediator regulation of TRP channels	0.044					
African trypanosomiasis	0.045					
Ras signaling pathway	0.047					
Phospholipase D signaling pathway	0.048					
Proteoglycans in cancer	0.049					

## Discussion

In this study, we applied high-throughput sequencing and bioinformatic analyses to characterize the transcriptional profile and epigenetic landscape of osteoclastogenesis in cells derived from female and male mice. Throughout this study, we focused our analyses on differences related to sex and differentiation state with a goal of identifying novel genes and pathways that may influence osteoclast differentiation in a sexually divergent manner. Our initial analyses correctly identified well-established genes involved in osteoclast function as well as known sexually divergent genes such as XIST (overexpressed in XX cells) and Uty (found only on Y chromosomes). That our unbiased analyses correctly identified anticipated variation at both the transcriptional and epigenetic level supports their ability to identify novel differences as well.

To manage the large datasets produced in this study, we utilized iPathwayGuide, which applies Impact Analysis to assign genes to pathways described by the Kyoto Encyclopedia of Genes and Genomes (KEGG) and statistically evaluates pathways according to fold changes and *p*-values of differentially expressed genes (Tarca et al., 2009; Ahsan & Drăghici, 2017). We deliberately selected low fold change thresholds for differentially expressed genes when performing pathway analyses for two reasons: first, there is evidence that small fold changes in gene expression can result in significant biological impacts and setting fold change thresholds too high can lead to disposal of potentially relevant genes, and second, by providing more genes to iPathwayGuide, the analysis was better equipped to evaluate pathway-level changes (St. Laurent et al., 2013). The top two sex-independent pathways altered by osteoclast differentiation, ranked by FDR-corrected *p*-value, were metabolic pathways and axon guidance. With respect to metabolic pathways, alterations were diverse with most changes falling within pathways of fatty acid metabolism, citric acid cycle, and oxidative phosphorylation. Almost without exception, when gene expression was altered in these pathways, it was increased. This concurs with prior findings of enhanced ATP production and mitochondrial activity in mature osteoclasts (Li et al., 2020; Da, Tao & Zhu, 2021). Some members of the axon guidance pathway have already been implicated in bone homeostasis such as the Eph/Ephrin and Slit/Robo axes (Matsuo & Otaki, 2012; Kim et al., 2018, p. 3; Jiang, Sun & Huang, 2022). In addition to identifying increased EphA, EphrinA, and EphrinB gene expression and decreased Slit1 expression in osteoclasts, we found altered expression of other axon guidance membrane receptors including Frizzled3 (Fzd3) and biregional cell adhesion molecule-related/down-regulated by oncogenes binding protein (Boc), which have not been directly interrogated for roles in osteoclast function.

Alterations in pathways associated with immune function was a common finding when comparing male and female-derived cells, which is in agreement with prior published findings (Gal-Oz et al., 2019; Mun et al., 2021). Interestingly, for multiple pathways linked to immune responses to diverse pathogens, expression of pattern recognition receptors is higher in male macrophages, but, after differentiation, female osteoclasts demonstrate higher expression. In the Toll-like receptor (TLR) pathway, for example, expression of all TLRs except TLR5 (for which there was no significant difference) was higher in female osteoclasts. By contrast, expression of TLR1, TLR2, and TLR9 was higher in male macrophages. These findings suggest that male naïve macrophages are more sensitive to TLR ligands and agree with prior findings of sexually dimorphic responses to inflammatory signals and induction of TLR expression in conjunction with pro-osteoclastogenic pathways (Marriott, Bost & Huet-Hudson, 2006; Li et al., 2009; Barcena et al., 2021; Chen, Lainez & Coss, 2021; Song et al., 2022). The potency of some TLR ligands, such as bacterial lipopolysaccharide (LPS), to stimulate osteoclastogenesis of osteoclast precursors pre-committed with RANKL is well established (Liu et al., 2009). It has also been shown that there is a sexually divergent response to LPS by differentiating osteoclasts with female cells demonstrating more robust differentiation (Mun et al., 2021).

In addition to pathways with previously established roles in osteoclast function, our analyses identified additional pathways that have not been studied directly in osteoclasts. For example, the Synaptic vesicle cycle (KEGG: 04721) was identified as a sex-independent up-regulated pathway in osteoclastogenesis. While this pathway has also appeared in analyses of differentially expressed genes in post-menopausal osteoporosis, roles of specific differential genes, such as Cplx2, Cacna1b, and Dnm1, have not been investigated (Zhu et al., 2018). This pathway and others not currently associated with macrophage or osteoclast function might represent the richest areas for discovery of novel gene functions, and we intend to investigate candidate genes of these pathways for deeper insights into osteoclast regulation.

As did the RNA-seq analyses, the ATAC-Seq studies identified expected sexually divergent chromosome accessibility with Y chromosome-specific loci overrepresented in male samples and X chromosome-specific loci overrepresented in female samples. The degree to which RANK signaling loosens chromatin is remarkable, with more accessible genes accounting for greater than 94% of the differences in both sexes. Given the balance of genes that are up- and down-regulated by RANK signaling, a similar pattern of chromatin accessibility might be expected. On the contrary, RANK signaling results in a more transcriptionally accessible epigenetic landscape. This might explain the ability of diverse inflammatory signals to complete osteoclastogenesis in RANKL-committed precursors—RANK signaling might render previously inaccessible pro-osteoclast genes available to transcription factors downstream from inflammatory receptors. By comparison, sex appears to have little influence in chromatin accessibility, suggesting that alterations in gene expression between male and female osteoclast precursors are driven primarily by transcriptional, rather than epigenetic, mechanisms.

Transcription appears to be a greater driver of osteoclastogenesis than epigenetic changes. In both female and male osteoclast precursors, pathway analysis revealed a greater number of significantly altered pathways following RNA-seq than ATAC-seq with minimal overlap between the two. Axon guidance was the only pathway with concordance between RNA-seq and ATAC-seq in both sexes, and while RNA-seq revealed a mixture of up- and down-regulated genes, ATAC-seq demonstrated an almost uniform increase in accessibility. While increased transcription in RANKL-treated cells may depend on increased chromatin accessibility, our data suggest that decreased transcription is not reliant on decreased chromatin accessibility.

## Conclusions

In this study we hypothesized that gene expression patterns and chromatin accessibility in osteoclast precursors are regulated by both RANK signaling and sex-specific factors. Using RNA-seq, ATAC-seq, and pathway analyses, we found that RANKL produces different transcriptional patterns in male- and female-derived cells, and these patterns suggest that male and female osteoclast precursors may respond differently to inflammatory signals. Furthermore, we found that RANK signaling results in a more permissive epigenetic landscape, with most loci becoming more accessible following treatment with RANKL. Multiple studies have demonstrated that inflammatory signals including LPS, poly I:C, and TNF can support the differentiation of committed osteoclast precursors while those same factors prevent osteoclastogenesis of naïve precursors. Our data suggest that male and female osteoclast precursors may have different sensitivities to these and other factors. Future studies should investigate these potential differences, which may further explain sexually divergent risk of bone loss.

## Supplemental Information

10.7717/peerj.14814/supp-1 Supplemental Information 1 Uncompressed −log padj volcano plots. These volcano plots depict data found in figures (A) 4, (B) 5, (C) 7, and (D) 8 with all −log adjusted *p*-values depicted.

10.7717/peerj.14814/supp-2 Supplemental Information 2 Altered transcriptional pathways during osteoclastogenesis.

10.7717/peerj.14814/supp-3 Supplemental Information 3 Altered transcriptional pathways between sexes.

10.7717/peerj.14814/supp-4 Supplemental Information 4 Differential chromatin accessible pathways during osteoclastogenesis.

10.7717/peerj.14814/supp-5 Supplemental Information 5 Differential chromatin accessible pathways between sexes.

10.7717/peerj.14814/supp-6 Supplemental Information 6 Differential transcriptional and chromatin accessible pathways during osteoclastogenesis in female cells.

10.7717/peerj.14814/supp-7 Supplemental Information 7 Differential transcriptional and chromatin accessible pathways during osteoclastogenesis in male cells.

10.7717/peerj.14814/supp-8 Supplemental Information 8 Differential expression in female macrophages and osteoclasts. Fold differences in gene expression with gene symbols, names, and statistics. Negative (−) values indicate greater expression in macrophages; positive values indicate greater expression in osteoclasts.

10.7717/peerj.14814/supp-9 Supplemental Information 9 Differential expression in male macrophages and osteoclasts. Fold differences in gene expression with gene symbols, names, and statistics. Negative (−) values indicate greater expression in macrophages; positive values indicate greater expression in osteoclasts.

10.7717/peerj.14814/supp-10 Supplemental Information 10 Differential expression between female and male macrophages. Fold differences in gene expression with gene symbols, names, and statistics. Negative (−) values indicate greater expression in male cells; positive values indicate greater expression in female cells.

10.7717/peerj.14814/supp-11 Supplemental Information 11 Differential expression in female and male osteoclasts. Fold differences in gene expression with gene symbols, names, and statistics. Negative (−) values indicate greater expression in male cells; positive values indicate greater expression in female cells.

10.7717/peerj.14814/supp-12 Supplemental Information 12 Differential peaks between female osteoclast precursor treated with MCSF+RANKL *vs* MCSF alone. Fold differences in chromatin accessibility with local genes and statistics indicated. Negative (−) values indicate greater accessibility in MCSF-only cells; positive values indicate greater accessibility in MCSF+RANKL cells.

10.7717/peerj.14814/supp-13 Supplemental Information 13 Differential peaks between male osteoclast precursor treated with MCSF+RANKL *vs* MCSF alone. Fold differences in chromatin accessibility with local genes and statistics indicated. Negative (−) values indicate greater accessibility in MCSF-only cells; positive values indicate greater accessibility in MCSF+RANKL cells.

10.7717/peerj.14814/supp-14 Supplemental Information 14 Differential peaks between female and male osteoclast precursors treated with MCSF alone. Fold differences in chromatin accessibility with local genes and statistics indicated. Negative (−) values indicate greater accessibility in male cells; positive values indicate greater accessibility in female cells.

10.7717/peerj.14814/supp-15 Supplemental Information 15 Differential peaks between female and male osteoclast precursors treated with MCSF and RANKL. Fold differences in chromatin accessibility with local genes and statistics indicated. Negative (−) values indicate greater accessibility in male cells; positive values indicate greater accessibility in female cells.

## Additional Information and Declarations

### Data Availability

The following information was supplied regarding data availability: The RNA sequencing data is available at NCBI GEO: GSE216929.
