# Supplementary figures and images for: RANK signaling in osteoclast precursors results in a more permissive epigenetic landscape and sexually divergent patterns of gene expression
Source: PeerJ. 2023 Feb 9;11:e14814. doi: 10.7717/peerj.14814 (PMC9922499; doi:10.7717/peerj.14814)

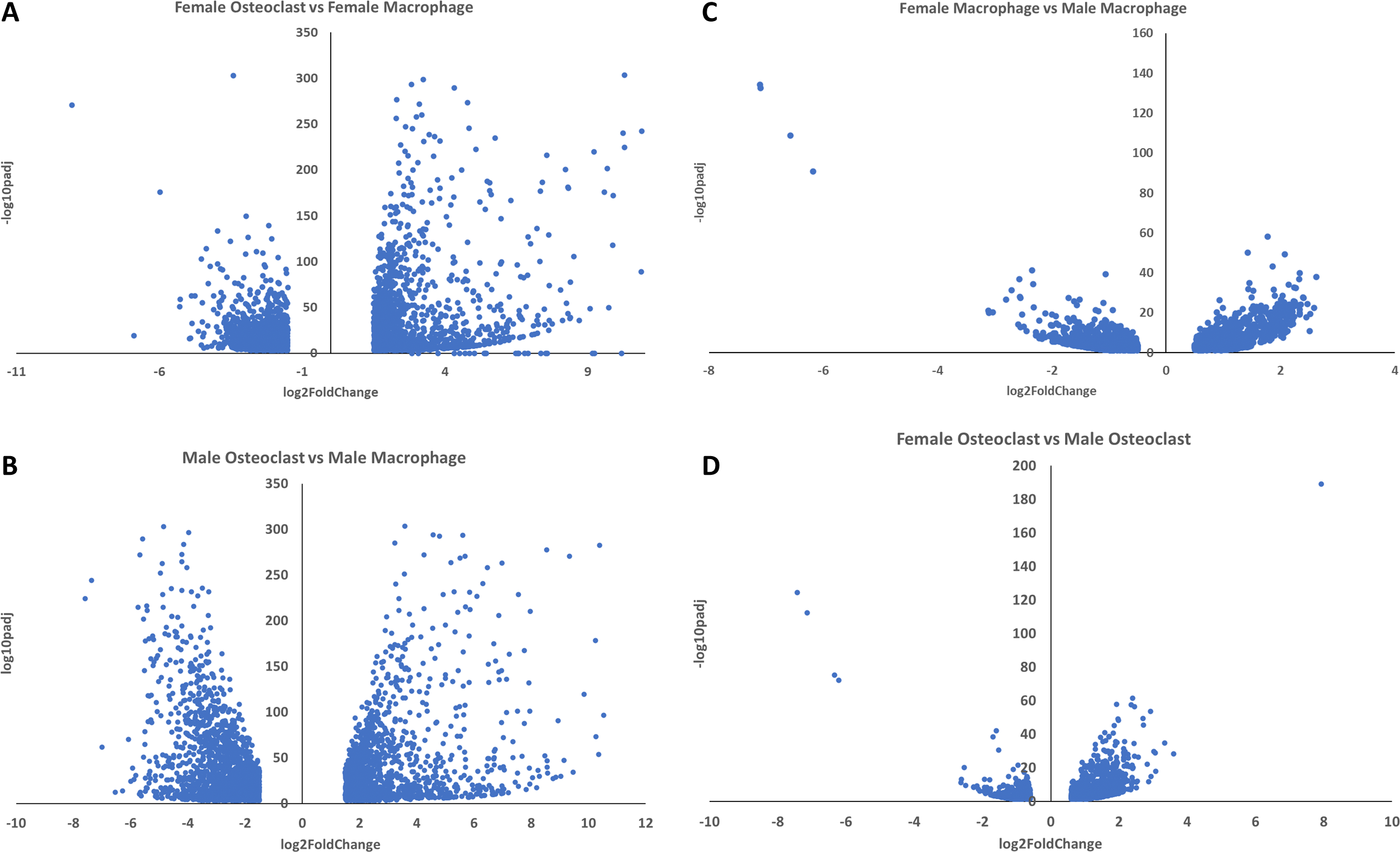

Supplement: Supplemental Information 1 — These volcano plots depict data found in figures (A) 4, (B) 5, (C) 7, and (D) 8 with all −log adjusted p-values depicted. [file peerj-11-14814-s001.png]
